# Body Odor Based Personality Judgments: The Effect of Fragranced Cosmetics

**DOI:** 10.3389/fpsyg.2016.00530

**Published:** 2016-04-18

**Authors:** Agnieszka Sorokowska, Piotr Sorokowski, Jan Havlíček

**Affiliations:** ^1^Smell and Taste Clinic, Department of Otorhinolaryngology, TU DresdenDresden, Germany; ^2^Institute of Psychology, University of WroclawWroclaw, Poland; ^3^Department of Zoology, Faculty of Science, Charles UniversityPrague, Czech Republic; ^4^National Institute of Mental HealthKlecany, Czech Republic

**Keywords:** body odor, olfaction, smell, personality assessment, cosmetics, perfume

## Abstract

People can accurately assess various personality traits of others based on body odor (BO) alone. Previous studies have shown that correlations between odor ratings and self-assessed personality dimensions are evident for assessments of neuroticism and dominance. Here, we tested differences between assessments based on natural body odor alone, without the use of cosmetics and assessments based on the body odor of people who were allowed to use cosmetics following their daily routine. Sixty-seven observers assessed samples of odors from 113 odor donors (each odor donor provided two samples – one with and one without cosmetic use); the donors provided their personality ratings, and the raters judged personality characteristics of the donors based on the provided odor samples. Correlations between observers’ ratings and self-rated neuroticism were stronger when raters assessed body odor in the natural body odor condition (natural BO condition; *r*_s_ = 0.20) than in the cosmetics use condition (BO+cosmetics condition; *r*_s_ = 0.15). Ratings of dominance significantly predicted self-assessed dominance in both conditions (*r*_s_ = 0.34 for natural BO and *r*_s_ = 0.21 for BO+cosmetics), whereas ratings of extraversion did not predict self-assessed extraversion in either condition. In addition, ratings of body odor attractiveness and pleasantness were significantly lower in natural BO condition than in BO+cosmetics condition, although the intensity of donors’ body odors was similar under both conditions. Our findings suggest that although olfaction seems to contribute to accurate first impression judgments of certain personality traits, cosmetic use can affect assessments of others based on body odor.

## Introduction

Fragranced cosmetics can affect the way people are perceived by others, and this effect has been observed in several contexts. Fragrances have been shown to influence perceptions of attractiveness (Baron, 1981; Dematte et al., 2007). In the latter study, the authors provide evidence that faces were rated as significantly less attractive when presented with an unpleasant ambient odor in comparison to the no-odor condition. Marinova and Moss (2014) showed that the use of gender-congruent fragrances can increase the “halo effect” of certain socially desirable characteristics, such as intelligence. Consequently, fragrances may also modulate self-perception, including self-confidence, which may in turn influence the attractiveness of the person wearing the fragrance. This effect has been demonstrated in previous studies using video footage in which persons wearing a pleasant fragrance were judged as more attractive than those who were not, despite the fact that raters could not perceive the odor (Higuchi et al., 2005; Roberts et al., 2009). Finally, there is some evidence suggesting that perfumes may affect impressions of people in professional contexts. For instance, Sczesny and Stahlberg (2002) showed that candidates using perfumes considered as typically masculine were perceived to be more suitable for a managerial position than those wearing a typically feminine perfume. However, Baron (1986) found that the effect of perfume on the impression conveyed by job applicants is modulated by other cues, such as their nonverbal behavior (perfumed applicants showing positive nonverbal cues were rated less positively by male interviewers than those with no perfume). Regarding the different genders, perfumed job candidates were evaluated especially favorably by female but not necessarily by male raters (Baron, 1983).

In Western cultures, natural body odor is generally perceived as unpleasant (Schleidt et al., 1981), and ratings of body odor pleasantness are on average relatively higher when participants use cosmetic products (Schleidt, 1980; Lenochová et al., 2012). Further, cosmetics may impede raters’ ability to discriminate individual body odor (Allen et al., 2015) or, based on body odor samples, discriminate between men and women (Schleidt, 1980); presumably because artificial odorants modify the impression conveyed by body odor intensity and pleasantness. Although it might seem that perfumes may “mask” or “cover” the underlying natural body odor, some studies proposed that fragrances could be enhancing body odor attractiveness in a complementary fashion (Milinski and Wedekind, 2001). Indeed, Lenochová et al. (2012) found that attractiveness ratings of perfume-body odor blends varied among individuals, suggesting that perfumes in fact interact with natural body odor rather than simply mask it. This is consistent with an observation that, compared with randomly assigned fragrances, the discrimination rates are higher when individual body odors are blended with fragrances that people choose for themselves (Allen et al., 2015).

Previous studies have shown that natural body odor may also play a role in impression formation (Havlicek et al., 2005; Sorokowska et al., 2012; Sorokowska, 2013a,b). Body odor can generate spontaneous attributions of personality traits, with unpleasant odors generally associated with socially undesirable traits (McBurney et al., 1976; Sorokowska, 2013b). A recent series of studies found that people were able to assess certain personality characteristics based on natural body odor samples and, that in some domains, these attributes were congruent with self-assessed traits of body odor donors. In the first of these studies, perception of extraversion, neuroticism, and dominance ratings based on body odor samples were higher than the chance level (Sorokowska et al., 2012). The results of the second study (Sorokowska, 2013a) showed that assessments based on body odor by both children and adults were congruent with self-report in the case of neuroticism. Additionally, adults were able to assess dominance above the chance level (Sorokowska, 2013a). The third study corroborated previous findings concerning accurate assessment of neuroticism and dominance from body odor alone (Sorokowska, 2013b).

Which mechanisms might possibly link personality traits to the body odor? First, human physiology and personality might overlap, as both are associated with certain hormones and neurotransmitters (Gray et al., 1991; Cashdan, 1995; Zuckerman, 1995). However, this relates mainly to neuroticism and dominance. Second, some emotions might be perceived from body odors (see e.g., Chen and Haviland-Jones, 2000; Ackerl et al., 2002; for review see Fialová and Havlíček, 2012) and hence influence the body odors of people who often experience these emotions (Dalton et al., 2013; see Sorokowska et al., 2012 for a Discussion). For example, repeatedly, emotionally induced sweating resulting from elevated anxiousness and nervousness might modify the body odor of neurotic people. Previous studies indicated that judgments of agreeableness, openness to experience, and conscientiousness were not congruent with the self-assessed traits of odor donors (Sorokowska et al., 2012; Sorokowska, 2013a,b). This might be because no direct hormonal links between body odor and these traits exist. Further, conscientiousness, agreeableness, and openness to experience seem not to be closely related to emotions influencing the body odor composition. As it was suggested in one of the previous papers (Sorokowska, 2013a), it is possible that people might need more context-dependent information to accurately assess these characteristics.

The studies reviewed in previous paragraphs tested the effect of fragrance use on sex discrimination or attractiveness judgments. However, no study has examined whether fragrances affect personality attributions based on odor cues yet. Thus, the main aim of our study was to test the effect of cosmetics use on personality attributions. We also aimed to extend previous findings related to assessments of attractiveness, odor intensity, and pleasantness of natural body odor relative to a body odor–fragrance blend. To do so, we asked a panel of raters to assess neuroticism, extraversion, and dominance of others based on the samples of natural body odor and body odor collected from participants using cosmetics. Based on previous findings, we hypothesized that assessments of neuroticism (a characteristic typically considered socially undesirable) would better predict self-assessed neuroticism in the natural body odor condition than in the cosmetics use condition. In contrast, we predicted no significant differences between the two conditions in ratings of extraversion and dominance.

## Materials and Methods

### Participants

#### Odor Donors

Odor donors were 113 individuals – 58 women aged between 17 and 33 years (*M* = 23.17, *SD* = 3.0) and 55 men aged between 20 and 34 years (*M* = 24.58, *SD* = 3.81). All donors provided informed consent prior to their inclusion in the study. They received a small gift (a set of cosmetics) for taking part in the study.

#### Odor Raters

Our rater sample comprised of 68 female students aged between 19 and 32 years (*M* = 22.88; *SD* = 2.16). None of the participants smoked or reported any olfactory-related impairment. Following previous work (e.g., Sorokowska, 2013a), we did not control for menstrual cycle phase or contraception use. The study was conducted in accordance with the Declaration of Helsinki, and all aspects of the study were approved by the Institutional Review Board at the University of Wroclaw. All raters provided informed consent prior to their inclusion in the study. They received a small gift (a cosmetic product) for their involvement.

### Procedure

#### Body Odor Sampling

We used armpit cotton pads to collect the body odor samples from odor donors. Such samples are less subject to possible odorous environmental contaminations relative to other methods (for details of the method see Sorokowska, 2013a). Body odor samples were collected twice from each donor: (i) without the use of cosmetics (i.e., natural body odor sample – natural BO condition) and (ii) while using cosmetics (BO+cosmetics). The odor donors were provided with two experimental sets each consisting of two 7 cm × 10 cm, 100% cotton pads, surgical hypoallergenic tape, unscented soap, a sterile 500 ml glass jar, and a new t-shirt.

For the collection of natural body odor samples, donors were asked to wash themselves with the unscented soap the morning of the experiment to attach the cotton pads under their arms with the surgical tape, put on the provided t-shirt (to avoid potential odor contamination from other clothes), and to wear the pads for twelve hours that day (Havlíček et al., 2011). The participants were asked to refrain from using scented cosmetics (e.g., fragrances, deodorants, and soaps), from consuming odorous foods (e.g., garlic, onions, or other spicy/odorous foods), and from drinking alcohol or smoking, beginning the day prior to the experiment (a standard procedure of studies that involve body odor assessment; e.g., Kohoutová, 2012; Roberts et al., 2013). Procedural instructions were provided in person and on a special instruction sheet that also included a questionnaire concerning the individual’s activity during the body odor collection. No participant reported any major deviations from the procedure.

After 12 hours, the participants placed the pads in jars and returned them to the experimenter. The samples were then frozen overnight. Freezing of such samples has been shown to have no significant impact on perceived body odor quality (e.g., Roberts et al., 2008; Lenochova et al., 2009).

A similar procedure was repeated for the second collection of body odor samples. However, in this case, participants were free to use scented cosmetics.

#### Personality Assessment

After providing body odor samples, donors completed a self-description TIPI-PL personality questionnaire (Gosling et al., 2003; Polish adaptation by Sorokowska et al., 2014). The TIPI-PL is based on the Big Five personality model (Extraversion, Neuroticism, Openness to experience, Conscientiousness, and Agreeableness), and it consists of 10 pairs of adjectives, 2 pairs for each Big Five dimension (for example, Extraversion: “Extraverted, enthusiastic” and reversed “Reserved, quiet”). Our main motivation to use the brief personality assessment was to maintain the same procedure for the odor donors and odor raters in our study. We added two questionnaire items to assess self-perceived dominance. Participants were asked to rate how much they thought each scale applied to them on a 7-point scale (where 1 = definitely disagree and 7 = definitely agree).

#### Statistical Analyses

In the main experiment, we first run series of *t*-tests to compare the average ratings based on left- and right-sided samples for both men and women. To test the effect of sex and condition (natural vs. BO+cosmetics condition) on body odor assessment, we computed repeated measures ANOVAs. Ratings did not follow a normal distribution, however, ANOVA is robust to normality violation when employed on a sample size of *N*> 100. Therefore, we employed the parametric test. The study used a 2 (sex of the odor donor) × 2 (natural BO vs. BO+cosmetics conditions as repeated measure) design. Analyses were performed separately for each personality trait.

The congruence between self-assessments and ratings based on body odor was calculated in two ways. First, to test whether the congruence was higher for natural body odor samples or cosmetics use samples, we computed a “deviation from congruence”, which was defined as the absolute difference between the self-assessment and rating (e.g., if self-assessed dominance was 5 and the rated dominance was 7, the “deviation from congruence” was 2). The lower the “deviation from congruence”, the higher the congruence between the self-assessments and ratings based on odors. Second, we compared Spearman correlation coefficients for natural vs. cosmetics use odor samples. We used Spearman ranks because, according to the Shapiro-Wilk test of normality, none of the self-assessed traits were normally distributed (all *p*s < 0.05).

We tested the effects of sex and condition (natural BO vs. BO+cosmetics) on congruence of assessments using a repeated measures ANOVA. In the experiment, a 2 (sex of the odor donor) × 2 (natural BO vs. BO+cosmetics conditions as repeated measure) design was employed. The analysis was again performed separately for each personality trait.

### Rating Sessions

#### Pilot Study

Methods used in previous studies of personality assessment based on body odor involved consecutive ratings of several personality characteristics based on a single odor sample (Sorokowska et al., 2012; Sorokowska, 2013a,b). Although this method decreases the possibility of olfactory adaptation, such a procedure may be more prone to the “halo effect”, in which raters’ assessments of various traits may not be entirely independent of one another (Nisbett and Wilson, 1977). Thus, prior to conducting the rating sessions, we tested for the possible presence of the “halo effect” by comparing two different procedures. In the first procedure, a group of 28 female judges (aged 19–22) assessed traits of a subset of odors “one by one”, i.e., each odor sample was rated for perceived Neuroticism, Extraversion, Agreeableness, and Dominance using a single answer sheet. In the second procedure, a different group of 28 female raters (aged 19–22) assessed each characteristic on a separate answer sheet (the judges first assessed the Neuroticism of all donors, then Extraversion of all donors, etc.; the sequence of samples was randomized). Each rater in our pilot study assessed 7 samples following one of the two procedures described above. In total, 49 samples of donors of both sexes were assessed. The samples that were used in the pilot study were not used in the main study (for example, if we used a sample from a right armpit of a given subject in the pilot study, in the main study we used a sample from the left armpit of the subject).

In the first procedure (consecutive assessments of traits), we observed significant correlations between rated Dominance and Neuroticism (*r*= 0.32, *p*= 0.03), Dominance and Extraversion (*r*= 0.37, *p* = 0.01), Dominance and Agreeableness (*r* = -0.48, *p* < 0.001), and Agreeableness and Neuroticism (*r*= -0.43, *p* = 0.002). In the second procedure (traits assessed separately), we observed a very similar pattern of correlations between Dominance and Extraversion (*r* = 0.58, *p* < 0.001), Dominance and Agreeableness (*r* = -0.46, *p* < 0.001), Agreeableness and Neuroticism (*r* = -0.43, *p* = 0.002), and Agreeableness and Extraversion (*r* = -0.35, *p* = 0.002). These correlations did not differ significantly between the two conditions for any of the traits assessed (test for difference between two correlation coefficients, *Statistica* software). Thus, we conducted the main study using consecutive assessments of traits. The main advantage of this procedure is that it is considerably less prone to olfactory adaptation as well as fatigue.

#### Main Experiment

In the main experiment, female raters were told to imagine a person connected to the scent they smelled, to rate his or her personality traits using a 7-point bipolar scale (the same which the donors had used to describe themselves), and to assess the sex of the person from whom the odor was taken (male/female). Following personality ratings, the judges rated the samples again, this time assessing the intensity, attractiveness, and pleasantness of the odor. Each woman rated the samples of six randomly selected odor donors (six samples of natural body odor and 6 samples of body odor with cosmetic use, both collected from the same odor donor).

## Results

### Subjective Perceptual Differences: Effect of Condition and Sex

We found no significant differences between the average ratings based on the right- and left-sided samples for neither men nor women (all *p*s > 0.05). However, male odors were rated as more intense [*F*(1,107) = 7.2, *p* < 0.008, $\eta_{p}^{2}$ = 0.06], more pleasant [*F*(1,106) = 7.0, *p*= 0.009, $\eta_{p}^{2}$ = 0.06], and marginally more attractive [*F*(1,106) = 3.1, *p* = 0.052, $\eta_{p}^{2}$ = 0.03] than were female odors. Additionally, we found sex differences in attributed psychological traits. Men were rated as less Agreeable [*F*(1,106) = 10.1, *p* < 0.002, $\eta_{p}^{2}$ = 0.09], more Neurotic [*F*(1,107) = 4.2, *p* < 0.05, $\eta_{p}^{2}$ = 0.04], and more Dominant [*F*(1,103) = 5.6, *p* = 0.02, $\eta_{p}^{2}$ = 0.05] than were women. For assessments of Extraversion, the effect of donor sex was only marginally significant [*F*(1,106) = 3.6, *p* = 0.06, $\eta_{p}^{2}$ = 0.03].

**FIGURE 1:**
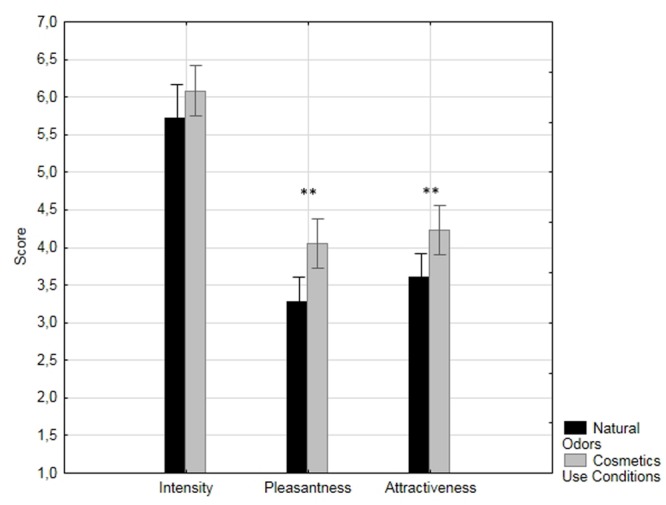
**Ratings of intensity, pleasantness, and attractiveness of body odor in the natural and cosmetics use conditions.** Significant *p* < 0.001 is marked by ^∗∗^.

We also found a significant effect of condition (see **Figure 1**). Body odor samples in the BO+cosmetics condition were assessed as more pleasant [*F*(1,106) = 19.1, *p*< 0.0001; $\eta_{p}^{2}$ = 0.15] and more attractive [*F*(1,107) = 13.4, *p* < 0.001, $\eta_{p}^{2}$ = 0.11] than were natural body odors, but there was no difference between the conditions in ratings of odor intensity (*F*(1,107) = 2.7, *p* = 0.10; $\eta_{p}^{2}$ = 0.02).

Further, there were no significant differences between conditions in personality judgments of body odor [Agreeableness (*F*(1,107) = 0.6, *p* = 0.4, $\eta_{p}^{2}$ < 0.01]; Dominance (*F*(1,103) = 0.2, *p* = 0.60, $\eta_{p}^{2}$ < 0.01; Neurotism (*F*(1,107) < 0.01, *p* = 0.9, $\eta_{p}^{2}$ < 0.01); Extraversion (*F*(1,107) = 2.8, *p* = 0.10, $\eta_{p}^{2}$ = 0.025)]. However, for Extraversion, we observed an interaction effect [sex-by-condition: *F*(1, 107) = 8.1, *p* = 0.005, $\eta_{p}^{2}$ = 0.07]. Extraversion ratings were higher for body odors in the BO+cosmetics condition than for natural body odors but only for female donors (*p* = 0.007; *post hoc* test with Bonferroni correction).

### Congruence between Self-Assessments and Ratings Based on Body Odor Samples

We found no significant differences in congruence between the natural and cosmetic use odor conditions for Agreeableness [*F*(1,107) = 0.4, *p* = 0.50, $\eta_{p}^{2}$ < 0.01] and Extraversion [*F*(1,107) = 2.9, *p* = 0.09, $\eta_{p}^{2}$ = 0.03]. Congruence for Dominance and Neuroticism were significantly lower for cosmetics use body odor samples than for natural body odor samples [*F*(1,103) = 5.9, *p* = 0.02; $\eta_{p}^{2}$ = 0.05] and [*F*(1,107) = 6.9, *p* = 0.01; $\eta_{p}^{2}$ = 0.06; respectively].

Similarly to the previous analysis, correlations between self-assessments and ratings for Agreeableness were lower in the cosmetics use than natural condition (0.04 and -0.09, respectively). For Extraversion, the correlation increased from -0.12 to 0.10, for dominance, it decreased from 0.34 to 0.21, and for Neuroticism, it decreased from 0.20 to 0.15. However, none of these differences were statistically significant (two-tailed tests, all *p*s > 0.12). It is noteworthy that we replicated previous findings concerning congruence of self-assessments and assessments based on natural body odor for Neuroticism (*p* < 0.04) and Dominance (*p* < 0.001) and that ratings of dominance remained significantly congruent in the cosmetics use condition (*p* < 0.04; see **Table 1**).

**Table 1:** Correlations between self-assessed personality traits and body odor based personality judgments.

Personality trait	Natural condition	Cosmetics condition
	
	*r*_s_	*r*_s_
Agreeableness	0.04	-0.09
Neuroticism	0.20^∗^	0.15
Extraversion	-0.12	0.11
Dominance	0.34^∗∗^	0.21^∗^

**p < 0.01; *p < 0.05.

## Discussion

The main aim of the current study was to test whether cosmetic use affects odor-based personality attributions. We corroborated previous findings demonstrating congruent perception between self-assessments and ratings of dominance and neuroticism based on natural body odors. In line with previous work, assessments of other personality traits (agreeableness, extraversion) did not correlate with self-reports. Critically, our results demonstrate that when odor donors could use cosmetics, odor-based perceptions of their neuroticism no longer correlated with self-reports. In contrast, ratings of dominance significantly predicted self-assessed dominance in both the natural and cosmetics use conditions.

In agreement with the current findings, previous studies showed that neuroticism and dominance were relatively accurately assessed based on body odors (Sorokowska et al., 2012; Sorokowska, 2013a,b) and that dominance might influence the perception of body odor attractiveness (Havlicek et al., 2005). Thus, the effects reported for assessments of neuroticism and dominance from body odor appear to be robust, whereas assessments of extraversion are significantly associated with self-report in only one study (Sorokowska et al., 2012). The current study confirmed previous findings for natural body odor samples and further showed that ratings of dominance remain significant under more realistic conditions (i.e., when odor donors are permitted to follow their daily hygienic routine and use any cosmetics that they may normally use).

One may speculate about the contrasting effect of cosmetic use on neuroticism and dominance assessments. As the use of cosmetics appears to be a part of our self-presentation, people may use cosmetics in order to express themselves in a socially desirable manner. Personality traits vary in their social desirability, with neuroticism being considered rather undesirable in Western cultural settings (Konstabel et al., 2006). People may attempt to suppress neuroticism related cues with their fragrance choice. In contrast, people who tend to be dominant in social interactions might select perfumes that do not interfere with the personality impression based on their body odor. Perhaps they might even present themselves as being more dominant than they are in reality. Indeed, dominance cues appear to be a desirable characteristic of fragrances, and one that is frequently employed in advertisement of men’s perfumes (Toncar and Fetscherin, 2012).

Consistent with previous studies (e.g., Lenochová et al., 2012), we observed increased ratings of attractiveness and pleasantness of body odor in the cosmetics use condition. However, there was no difference in personality attributions (with the exception of extraversion ratings) between the cosmetics use samples and the natural body odors. Further, effect sizes (as assessed by partial eta squared) for the differences between the two conditions were quite low; suggesting that the effect of cosmetics use on mean values in personality attributions is rather modest.

There is robust evidence indicating that female body odor is on average considered more pleasant and less intense than male body odor is (McBurney et al., 1976; Hold and Schleidt, 1977). It also appears that more intense odors are stereotypically attributed as male, independent of the actual sex of the odor donor (Doty et al., 1978). Our study replicated past findings related to male body odor intensity, however – interestingly – we found that male body odors were rated as more pleasant than were female body odors. This result might be due to the fact that in our study, only female raters assessed the body odor samples. However, women are commonly found to be slightly more sensitive to various odors than men (Doty and Cameron, 2009), and they attach higher importance to olfaction in both sexual and non-sexual context (Havlicek et al., 2008). Also, previous studies regarding smell have shown that women more accurately recognize the sex of a donor on the basis of body odor (Hold and Schleidt, 1977) and that they are generally more accurate in their assessments of personality based on odor samples (Sorokowska et al., 2012). Although these are among the reasons that we employed female raters, future studies may test whether different results are obtained using male raters.

In the cosmetics use condition, participants were permitted to use fragranced cosmetics according to their personal routine. We did not control the quantity nor type of the cosmetics (i.e., deodorants, antiperspirants, perfumes) used by odor donors. The main rationale for this procedure was to collect the axillary odor samples under highly realistic conditions (i.e., to achieve high external validity). Also, Lenochová et al. (2012) showed that cosmetics selected by participants have higher effects on pleasantness and attractiveness ratings of body odor samples than do assigned cosmetics, which additionally suggests that assigned cosmetics might have differential effects on various body odor samples. In a similar line, it was recently reported that using your own fragrance compared to the assigned one increased success rate in individual discrimination of the fragrance-body odor blends (Allen et al., 2015). However, the procedure we used did not allow us to test the potential effect of different types of fragranced cosmetics. Thus, future studies should control for the type of cosmetics used by participants and investigate whether the cosmetics chosen by the participants compared to cosmetics assigned to them by researchers have different effects on how they are perceived. Future studies may also assess whether people are able to consciously modify the personality impression conveyed by the cosmetics they select. Finally, it would be of interest to examine whether different scents are chosen by participants depending on the social context (e.g., for a romantic meeting in contrast to a job interview).

It can be argued that some of the effects reported here might be attributed to the rating procedure. More specifically, raters were asked to assess all personality characteristics consecutively after smelling each odor sample. Although this procedure could potentially result in the “halo effect” (i.e., an impression made in one domain is transferred to an impression made in another domain in a stereotypic fashion; Nisbett and Wilson, 1977), the results of our pilot study indicated no major signs of the “halo effect” using this experimental paradigm for odor-based assessments. The main reason why we did not employ separate ratings of each trait is that it is considerably more time-consuming and, importantly, ratings might be affected by olfactory adaptation and fatigue. Another possible limitation of our study might be the use of the TIPI-PL scale both to measure the personality characteristics of the odor donors and to perform the ratings based on body odor samples. The TIPI is a very brief method (two items each consisting of two adjectives, i.e., four adjectives per personality characteristic), and its psychometric parameters are somewhat lower than those of longer inventories measuring the Big Five characteristics (Gosling et al., 2003; Sorokowska et al., 2014). However, thanks to the brevity of this tool, the raters could assess the samples using the same scales that the donors had used to describe themselves, and this enabled us to measure the congruence of self-assessment and odor-assessment sessions more precisely than in the case of the previous studies regarding the body odor and personality. Nevertheless, using the TIPI test could make both the self-assessments and ratings based on the odor samples slightly less reliable.

As discussed above, the congruent attribution of some personality domains based on body odor, namely neuroticism and dominance, appears to be robust. However, it is unclear whether people spontaneously employ these particular attributions when assessing others based on odor cues. Related research on personality attributions based on facial cues suggests that the most important dimensions are agreeableness and dominance (Oosterhof and Todorov, 2008). In the case of body odor, the hedonic dimension (pleasantness/attractiveness) and strength (intensity) seem to be among the most salient. As it was hypothesized in the previous studies (Sorokowska, 2013b), it is possible that the overall perceived pleasantness of odor samples might drive the personality-related judgments. However, it is also possible that some sex stereotypes might be additionally involved in this process, given that, like in our research, male and female body odor samples are generally rated differently. Additionally, research shows that unpleasant body odors are often associated with typically male characteristics (McBurney et al., 1976), which might create another link between sex stereotypes and these attributions. To understand the underlying cognitive processes related to personality assessments based on body odors, future studies should focus more on the overall impressions created by odor samples and investigate spontaneous associations generated by these odors.

To summarize, the current study tested the effect of cosmetic use on personality attributions. Our results showed that, when judging personality based on body odors of people using cosmetics, the raters were able to accurately assess the odor donor’s dominance but not neuroticism. It seems that cosmetics bias assessments of some important social cues and allow people to modify the impression they convey. People may employ cosmetics to be perceived in a socially desirable fashion and may attempt to cover cues that can lead to socially undesirable perception such as neuroticism. Future studies should explore how different types of cosmetic products such as deodorants and various perfumes specifically affect odor based personality judgments.

## Author Contributions

All authors conceived and designed the study. AS collected the data. AS and PS analyzed the data. All authors drafted, critically revised, and approved the final version of the manuscript.

## Conflict of Interest Statement

The authors declare that the research was conducted in the absence of any commercial or financial relationships that could be construed as a potential conflict of interest.
